# Plants under Siege: Investigating the Relevance of ‘*Ca*. P. solani’ Cixiid Vectors through a Multi-Test Study

**DOI:** 10.3390/plants12244157

**Published:** 2023-12-14

**Authors:** Andrea Kosovac, Emil Rekanović, Živko Ćurčić, Jelena Stepanović, Bojan Duduk

**Affiliations:** 1 Institute of Pesticides and Environmental Protection, 11080 Belgrade, Serbia; emil.rekanovic@pesting.org.rs (E.R.); jelena.stepanovic@pesting.org.rs (J.S.); bojan.duduk@pesting.org.rs (B.D.); 2 Institute of Field and Vegetable Crops, 21000 Novi Sad, Serbia; zivko.curcic@ifvcns.ns.ac.rs

**Keywords:** stolbur phytoplasma, *Hyalesthes obsoletus*, *Reptalus quinquecostatus* Dufour, epidemiology, sugar beet, maize, tobacco

## Abstract

Crop losses caused by the plant pathogenic bacterium ‘*Candidatus* Phytoplasma solani’ (CaPsol) underscore the need to better understand its perplexing epidemiological pathways. *Hyalesthes obsoletus* (Hemiptera, Cixiidae) is a prominent CaPsol vector with three plant associations in Serbia (*ex Urtica dioica*/HobsUd; *ex Convolvulus arvensis*/HobsCa; *ex Crepis foetida*/HobsCf). Another cixiid planthopper, *Reptalus quinquecostatus* (Dufour), has been recently confirmed as a noteworthy CaPsol vector. A multi-test study assessed the relevance of *H. obsoletus* associations and *R. quinquecostatus* populations from *Crataegus monogyna* and *Prunus spinosa* in CaPsol occurrence in sugar beet, maize, and tobacco. Molecular typing of the CaPsol strains transmitted to test plants in experimental trials provided the first evidence of HobsUd transmitting CaPsol tuf-a type to sugar beet, HobsCa infecting maize and tobacco with tuf-b type, and HobsCf transmitting CaPsol tuf-b to maize. Affiliation of *R. quinquecostatus* with the specific CaPsol genotype, dSTOLg, was reaffirmed in this study. The possible involvement of *R. quinquecostatus* in maize redness disease and tobacco stolbur was suggested, given that this cixiid was identified as a vector of CaPsol to these crops. The obtained results indicate that the tested vectors pose a threat to cultivated plants in Serbia, underscoring the need to recognize their relevance in CaPsol disease occurrences.

## 1. Introduction

‘*Candidatus* Phytoplasma solani’, a plant pathogenic bacterium classified under the Mollicutes class, is commonly referred to as stolbur phytoplasma [1]. The infection of plants by this pathogen manifests in various symptoms, including leaf yellowing (chlorosis), stunted growth, irregular leaf and flower development, fruit deformities, etc. [2]. Abnormalities in plant physiology and morphology induced by ‘*Ca*. P. solani’ (CaPsol) can have an agroeconomic impact on numerous cultivated plants such as potato (30–80% yield losses and reduced seed quality), tomato (losses of up to 60%), grapevin (80%), peppe (90%), maize (variable losses of 10–90%), celery (100%), sugar beet (up to 100%), etc. [3,4,5,6,7,8].

Plant diseases associated with ‘*Ca*. P. solani’ exhibit a recurrent occurrence pattern and a complex epidemiology, with only a limited number of insects from the suborder Auchenorrhyncha (Hemiptera) identified as vectors, notably prominently within the family Cixiidae (Fulgoromorpha) [9]. Vectors introduce CaPsol into crop fields either as CaPsol-infected intruders originating from natural vegetation, or by emerging from weed plants within or in the vicinity of fields that act as CaPsol infection inoculum sources. In the case of mono/oligophagous vectors, the transmission of phytoplasma occurs through their erroneous feeding on crop plants, while polyphagous vectors transmit the pathogen as a result of their indiscriminate feeding behavior [9]. Another important ecological aspect of CaPsol epidemiology involves vectors that have undergone a host shift, completing their lifecycle within crop rotation systems and leading to the pathogen persistently remaining within a limited host range [10,11].

The principal CaPsol vector in the Euro-Mediterranean region is the cixiid planthopper *Hyalesthes obsoletus* Signoret. This insect, associated with specific plants of natural vegetation, disseminates two biologically distinct groups of CaPsol strains, tuf-a and tuf-b, designated based on the genotyping of the *tuf* gene (encoding elongation factor Tu) [12,13,14,15]. The populations of *H. obsoletus* associated with *Urtica dioica* L. (a vector further referred to as HobsUd) are responsible for transmitting CaPsol strains of the tuf-a type that encompasses the tuf-a and tuf-b2 genotypes [13,14,16,17]. Recently, a tuf-a type CaPsol infection in a crop plant was reported in Serbia, affecting sugar beet [8]. *H. obsoletus* populations associated with *Convolvulus arvensis* L. (HobsCa) are responsible for outbreaks of several CaPsol-associated diseases in Serbia belonging to the tuf-b type, in some cases with pathogen strains identified as the tuf-b1 genotype [15,18,19]. Lastly, the *H. obsoletus* populations associated with the plant *Crepis foetida* L. (HobsCf) in Serbia aid in the spread of tuf-b type CaPsol strains, and notably, the host, *C. foetida*, can harbor both the tuf-b1 and tuf-d CaPsol genotypes [15,20].

Sugar beet (*Beta vulgaris* L.) has been reported as susceptible to a plant disease of unknown etiology in the second half of the 20th century, characterized by leaf wilting and a distinctive rubbery texture of the taproot [21]. Since 2018, there have been outbreaks of Rubbery Taproot Disease (RTD) in sugar beet in northern Serbia and it has been reported in other countries in the Pannonian Plain, with a confirmed association with CaPsol [7,8]. An RTD case study in Serbia revealed that CaPsol tuf-b epidemiological pathways were responsible for the disease occurrence [19]. Cixiid planthopper *Reptalus quinquecostatus* (Dufour) *sensu* Holzinger et al. [22] emerged as the vector responsible for the in situ RTD outbreak associated with the specific tuf-d CaPsol genotype, dSTOLg [19]. The abundant population of *R. quinquecostatus* that was found in the sugar beet field could not be associated with this crop plant since nymphs were not found on its roots [19]. While the dSTOLg CaPsol strains were identified in various weed plants hosting *R. quinquecostatus* adults [19], the complete epidemiological cycle of the RTD disease remained incompletely clarified, leaving uncertainties regarding the CaPsol infection source for this vector and its host plant(s). In contrast to HobsCa, which was identified as a vector for the CaPsol tuf-b type to sugar beet [19,23], HobsUd was reported as incapable of transmitting the tuf-a-type CaPsol to this crop plant [23]. The importance of addressing the question regarding the vectoring potential of HobsUd was highlighted with findings of the tuf-a/b2 CaPsol genotypes in sugar beet in Serbia [8].

Maize (*Zea mays* L.) has been documented in Serbia since the mid-20th century as being susceptible to a disease characterized by reddening of the leaf midrib, lamina, and stalks, as well as abnormal ear development and a reduced seed yield [24]. In the early 2000s, the maize redness disease was associated with CaPsol of the tuf-b type and a cixiid planthopper with a lifecycle adapted to maize/wheat crop rotation—*Reptalus panzeri* (Löw) [11,25]. *R. quinquecostatus* (Dufour) was also recorded in CaPsol-affected maize fields; however, as its populations tested negative for CaPsol, its relevance in spreading maize redness was not further evaluated [25]. The presence of *R. quinquecostatus* populations on maize was confirmed on multiple sites across northern Serbia in 2022, highlighting the importance of reassessing its role in the CaPsol epidemiology in the context of maize redness disease in Serbia [26]. Given that the CaPsol tuf-b type has been documented in maize [27], it questions the potential involvement of HobsCa and HobsCf, both known vectors of the tuf-b type, in the transmission of CaPsol to this crop plant. 

While the etiology of the aforementioned sugar beet and maize diseases was recently associated with CaPsol, tobacco (*Nicotiana tabacum* L.) was reported as affected by ‘tobacco stolbur’ disease half a century ago, with symptoms such as infertility of female flowers, severe wilting, and drying of leaves, which tend to rot when collected in bales [28]. At that time, the suggested transmitter of the disease to tobacco plants was HobsCa, which had already been experimentally confirmed as a vector of ‘stolbur’ [29,30]. CaPsol infection in tobacco plants has been reported in Serbia in the modern-day [31], but since then, there have been no additional data regarding the occurrence of the disease or the vectors involved. Better understanding tobacco’s susceptibility to CaPsol is an important issue, as Serbia is the sixth largest tobacco producer in Europe, with a growing production trend and an area of 5803 ha that was covered with this crop in 2021 [32].

The conducted multi-test study assesses the vectoring relevance of the two cixiid vectors *H. obsoletus* and *R. quinquecostatus*, associated with specific natural vegetation hosts, in the occurrence of CaPsol-associated diseases in three economically important plants: sugar beet, maize, and tobacco. Through a series of experimental transmission tests and genotyping of CaPsol strains on three epidemiologically informative genes: *tuf*, *stamp* (encoding an antigenic membrane protein) and *vmp1* (encoding a variable membrane protein), our research aims to enhance the understanding of CaPsol disease occurrence in Serbia, offering insights that hold broader significance within the European context. Tests with sugar beet involved (1) HobsUd association to confirm its ability to transmit CaPsol tuf-a and (2) *R. quinquecostatus* populations from the two plants, *Crataegus monogyna* Jacq. (further referred to as RqCm) from an undisturbed habitat and *Prunus spinosa* L. (RqPs) from arable land borders, to verify the vector’s potential and preference for the dSTOLg CaPsol genotype. Maize test plants were exposed to the (1) tuf-b type vector HobsCa, (2) the HobsCf population sampled in the maize field vicinity, and (3) RqPs to reassess the role of this cixiid in the maize redness disease. The tobacco experimental plants were exposed to (1) HobsCa and (2) *R. quinquecostatus* (RqCm and RqPs) to evaluate their role as CaPsol vectors. While *H. obsoletus* does not complete its life cycle on crop plants, a trait that may also be currently attributed to *R. quinquecostatus* based on the present knowledge of its biology, these insects continue to pose a threat to cultivated plants and should be recognized as potential contributors to CaPsol disease outbreaks.

## 2. Results

### 2.1. Species Identification and CaPsol Infection in Cixiid Populations

In the collected insect samples from all vector populations, species identification was carried out using a representative sample size of 30 individuals per population. The morphological analysis confirmed the identity of *H. obsoletus* specimens across all three associations (HobsUd, HobsCa, and HobsCf). For *Reptalus* sp. individuals sampled from the three populations (RqCm, RqPs1/locality Botoš, and RqPs2/locality Plavi Horizonti), all individuals were identified as *R. quinquecostatus sensu* Holzinger et al. [22]. This identification was supported by a morphological analysis of male genitalia, which revealed a distinct left-oriented process in all male individuals. A species-specific molecular procedure based on the internal transcribed spacer 2 (ITS2) gene region confirmed the identity of all female specimens as *R. quinquecostatus*. CaPsol infection rates of *H. obsoletus* populations showed variability, ranging from the lowest at 10% in HobsCf (3/30) and 30% in HobsUd (9/30) to 42% in HobsCa (13/30). In contrast, *R. quinquecostatus* populations exhibited higher CaPsol infection rates than *H. obsoletus*, ranging from 50% in RqPs1 to 83% in the RqCm population, with RqPs2 at 80%.

### 2.2. Evaluation of CaPsol Transmission Efficacy

**Figure 1:**
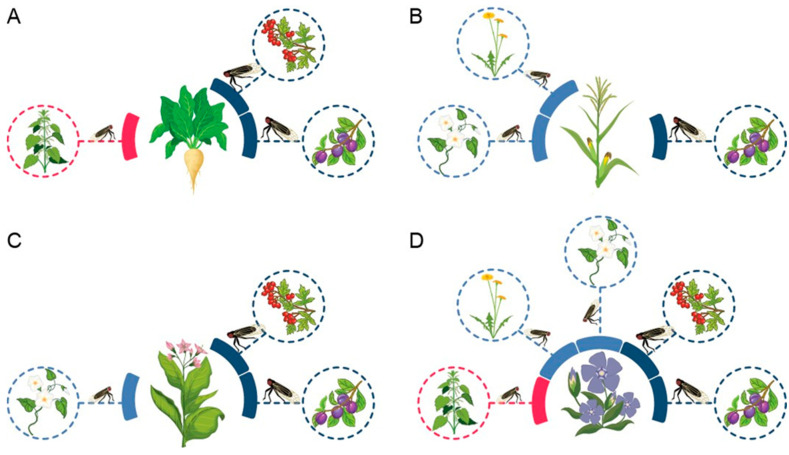
CaPsol transmission multi-test involving sugar beet (**A**), maize (**B**), tobacco (**C**), and the experimental plant periwinkle (**D**). Populations of specific cixiid vectors associated with the respective host plant are depicted in the surroundings of each plant. Red lines indicate tuf-a CaPsol epidemiological route including HobsUd, light blue lines depict tuf-b CaPsol routes vectored by HobsCa and HobsCf, and dark blue lines correspond to RqCm and RqPs populations transmitting tuf-d type CaPsol.

Through successful trials in a multi-test setup, the capacity of all tested vector populations to transmit CaPsol to the corresponding experimental plant species was confirmed (Figure 1; Table 1).

Tests with sugar beet plants demonstrated that the HobsUd association can transmit tuf-a and tuf-b2 CaPsol genotypes to this crop plant, achieving a 27% infection success rate (Table 1). The multilocus CaPsol genotypes transmitted to sugar beet by HobsUd were tuf-a/SB5/V3 and tuf-b2/RTD6/V23, while in periwinkle plants, tuf-b2/19-25/V18 and tuf-b2/RTD6/V23 were detected. *R. quinquecostatus* was confirmed as an effective vector of the dSTOLg genotype (tuf-d/STOL/V2-TA) to sugar beet, with only one STOLg (tuf-b/STOL/V2-TA) strain being transmitted by the RqPs1 population. The RqCm population had a lower transmission success rate, with 1/5 plants infected with a new *stamp* genotype (St96).

Almost half of the maize plants exposed to the RqPs2 population were successfully infected with CaPsol. The multilocus genotype STOLg was fully characterized, two STOL strains could not be typed on the *tuf* and *vmp1* genes, and a new *stamp* genotype (St101) was transmitted. The HobsCa population was equally successful in infecting maize plants (40%), with the STOLg strain transmitted, along with Rqg31 and Rqg50 genotypes and three new *stamp* genotypes (St97, St98, and St99). The HobsCf population also succeeded in infecting maize plants and transmitting the STOL genotype, Rqg31, and another new genotype (St100), although these strains remained incompletely characterized on genes other than the *stamp*.

Tobacco plants exhibited a high susceptibility to CaPsol transmitted by the tested populations of both vector species. HobsCa achieved a transmission success rate of 80%, while *R. quinquecostatus* showed a transmission rate ranging from 40% to 60% (Table 1). The HobsCa population transmitted predominantly the tuf-b/Rqg31 genotype associated with V2-TA, V4, or V14 type (6/8 infected plants); tuf-b/Rqg50/V14; and a new *stamp* genotype (St102). In contrast, *R. quinquecostatus* populations from different plants transmitted both dSTOLg and STOLg genotypes, as well as tuf-b/Rqg31/V2-TA.

Periwinkle plants, which served as positive controls to assess the diversity of CaPsol strains in vector populations, showed that the tested HobsCa population is capable of transmitting the dSTOLg genotype, whereas HobsCf exclusively transmitted tuf-b type CaPsol strains. All three of the *R. quinquecostatus* populations showed a high efficiency in transmitting dSTOLg, followed by tuf-b/STOL and tuf-b/Rqg50 (Table 1).

### 2.3. Revealed Diversity of CaPsol Strains Based on the Stamp Gene 

**Figure 2:**
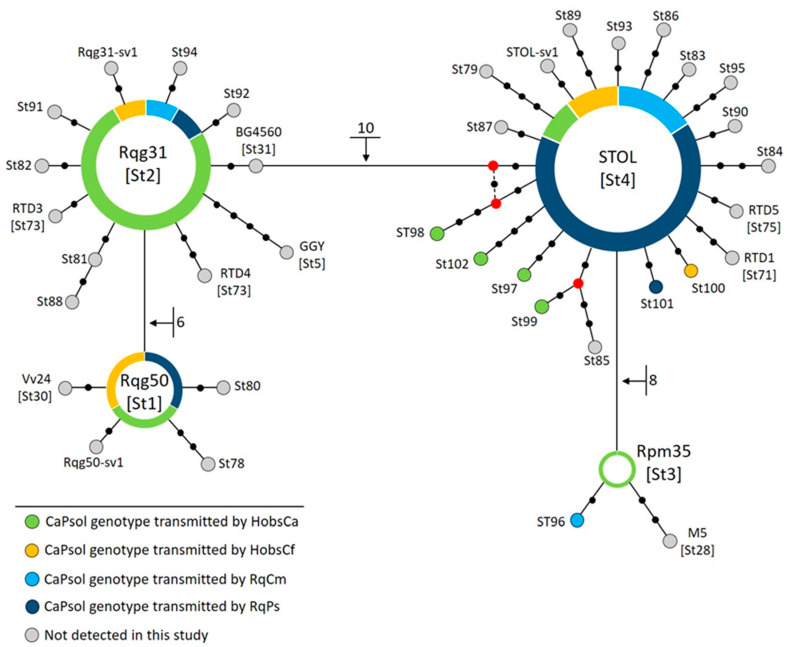
Median joining network constructed using CaPsol *stamp* genotypes associated with tuf-b and tuf-d types identified so far in Serbia [19,20,35]. Circles represent a specific CaPsol *stamp* genotype with ‘St’ *stamp* sequence variants provided in square brackets corresponding to the original *stamp* genotype designation [19,33,34]. The size of the circle indicates the frequency of the genotype in this research. Colors have been assigned according to insect vector populations, as described in the legend, and they are arranged proportionally within genotype circles based on the transmission results. The black dots on the connecting lines represent the number of mutations between linked genotypes, as do the numbers noted next to the connecting lines. The red dots in the network are median vectors representing missing or unsampled intermediate genotypes.

Genotyping of transmitted CaPsol strains using the *stamp* gene confirmed the basic epidemiological pattern. Specifically, tuf-a/b2 strains identified as SB5, 19–25, and RTD6 followed a consistent transmission route through HobsUd (Table 1). Conversely, CaPsol genotypes of tuf-b epidemiology (tuf-b and tuf-d types) were found to be transmitted through HobsCa, HobsCf, and all *R. quinquecostatus* populations (Table 1; Figure 2). The *stamp* genotype STOL/St4 prevailed in test plants, primarily due to its transmission with *R. quinquecostatus*, where 31/37 CaPsol isolates were attributed to this particular genotype. Of the 31 STOL strains transmitted by *R. quinquecostatus*, 24 were specifically linked to the tuf-d and V2-TA types within the distinct dSTOLg genotype. Among the other CaPsol *stamp* genotypes transmitted by *R. quinquecostatus* were Rqg31/St2 and Rqg50/St1, along with two previously unreported genotypes (St96 and St101). Genotype St96 exhibited a single nucleotide difference to Rpm35, while St101 differed by only one nucleotide from STOL (Figure 2). The HobsCa population also transmitted the STOL/St4 genotype, although, among them, two were of the tuf-b type, while one belonged to the dSTOLg genotype (tuf-d type). Notably, the majority of transmitted CaPsol strains was associated with the Rqg31/St2 genotype, accompanied by two strains of Rpm35/St3, two strains of Rqg50/St1, and four new genotypes, all diverging from the STOL/St4 genotype (Figure 2). In particular, the strain St102 differed by three nucleotides, while St97 exhibited a divergence of two nucleotides from STOL. Genotype St98 displayed more intricate relationships, as evidenced by a reticulation within the network, while St99 was found to be related to a previously described genotype St85 (Figure 2). The assessment of the HobsCf population reaffirmed its role as a vector of tuf-b type CaPsol strains, primarily transmitting the STOL/St4 genotype. HobsCf also transmitted Rqg50/St1, Rqg31/St2, and a novel genotype, St100, which is closely associated with STOL, differing by one nucleotide. 

## 3. Discussion

The applied experimental multi-test approach involved economically important plants susceptible to CaPsol that are exposed to tested cixiid vectors in their natural surroundings in Serbia. The obtained results offer valuable insights into the CaPsol routes through which the pathogen can be introduced into crop fields originating from plants not necessarily present as weeds within arable land. The capacity of *H. obsoletus* to transmit CaPsol was reaffirmed; HobsUd transmitted tuf-a strains to sugar beet and HobsCa infected maize and tobacco with tuf-b, while the risk of CaPsol-associated diseases linked to HobsCf was highlighted through infections of maize plants. *R. quinquecostatus* was reaffirmed as a significant CaPsol vector in sugar beet RTD through tested populations from natural and semi-natural habitats. Its role in other CaPsol diseases also opens a debate considering successful infection trials with maize and tobacco plants. The newly acquired information on the vectoring potential of *H. obsoletus* associations and *R. quinquecostatus* populations in Serbia revealed in this study broadens the list of CaPsol plants threatened by these vectors and adds complexity to the design of CaPsol management strategies.

A previous study on CaPsol epidemiology in sugar beet in Serbia was focused on tuf-b vectors [19]. However, tuf-a CaPsol strains found in this crop [8] raised the question of whether HobsUd could be responsible for its transmission, despite previous evidence suggesting its inability to transmit CaPsol to sugar beet [23]. Both CaPsol genotypes, SB5 and RTD6, previously found in sugar beet in Serbia [8] were successfully transmitted by HobsUd in the conducted tests. This suggests that HobsUd should be considered a potential threat in sugar-beet-growing areas on a broader European scale where *U. dioica* serves as a CaPsol inoculum source.

Although CaPsol-induced plant diseases have, in certain instances, been linked to vectors associated with crop plants [10,11], multiple reports highlight that HobsCa populations, associated with a non-crop plant, are the main contributors to CaPsol tuf-b type outbreaks in Serbia [15,18]. HobsCa completes its life cycle on *C. arvensis* plants present within arable land, while CaPsol-infected HobsCa populations have also been found on *C. arvensis* in ruderal and undisturbed habitats [36]. The results from the obtained transmission tests indicated the ability of HobsCa to introduce CaPsol infections into maize fields. Furthermore, experiments with tobacco plants underscore the potential of HobsCa for causing CaPsol infections in this crop plant. The prevalent CaPsol strains transmitted by HobsCa were of the Rqg31 *stamp* genotype, aligning with prior data from Serbia [15,18,19].

HobsCf, previously associated with the tuf-b type CaPsol epidemiology in grapevine (Bois Noir disease) [15], was affirmed in transmission trials as an effective vector of tuf-b type strains. While the tuf-d genotype was reported to infect its host plant, *C. foetida* [20], CaPsol strains of this type were not transmitted by the HobsCf population assessed in this study. However, this does not eliminate the possibility of this vector being involved in the dissemination of the tuf-d type/genotype, along with the currently prominent transmitter, *R. quinquecostatus*, and the confirmed but minor vector, HobsCa [19]. Moreover, multi-test results have shown that HobsCf can transmit CaPsol genotypes beyond the STOL genotype [15]. Transmission tests conducted with maize plants have revealed that HobsCf has a broader vectoring capacity, potentially endangering various other crop plants across the countries encompassed by its vast distribution area [37].

In contrast to the unveiled lifecycle and host plant associations of *H. obsoletus,* the endogeic life of *R. quinquecostatus* is unknown, with limited data from eastern Serbia reporting the presence of the nymphs in the rhizosphere of *Koeleria macrantha* (Ledeb.) Schult (*Poaceae*) [38]. In Western Europe, adults prefer moist habitats with scattered shrubs and tall herbs, and in Italy, populations of *R. quinquecostatus* were reported on *Ulmus pumila* L., while in Poland, adults have been found in herbaceous meadows [39,40,41]. In Turkey, adults were reported as favoring, amongst other plants, *Crataegus* sp. and *Prunus* spp. [42]. An *R. quinquecostatus* population in an RTD case study in Serbia preferred several weeds along the edges of a sugar beet field and had a decreased presence toward the center of the plot [19]. A similar behavioral pattern was observed in the case of CaPsol-affected maize fields, where *R. quinquecostatus* was observed mainly along the field border adjacent to the weed-covered area [11]. These observations suggest that this cixiid has entered arable land from the surrounding vegetation rather than originated within the crop field. *R. quinquecostatus* populations were observed from 2020 to 2022 across multiple sugar beet fields in Serbia, while sporadic occurrences were recorded on maize, tobacco, parsley, and other crops, sometimes in sympatry with *R. panzeri* [26,43]. The origin and sources of CaPsol infection for *R. quinquecostatus* populations in crops remain uncertain; nevertheless, its populations have been found on *C. monogyna* and *P. spinosa* shrubs in northern Serbia, with population infection rates exceeding 80%. Multi-test trials have confirmed that this CaPsol vector poses a significant threat to sugar beet, while its populations have been demonstrated for the first time to be a threat to other crops such as maize and tobacco, overall underlining a previously noted association with the dSTOLg genotype [19].

The epidemiology of CaPsol-induced diseases is perplexing because of the involvement of multiple vectors and diverse plant inoculum sources, presenting a challenge in developing effective control strategies. The management of phytoplasma diseases varies depending on the particular epidemic scenario, and involves the mechanical and chemical elimination of plant inoculum sources and the control of vectors through the application of insecticides [44]. Phytoplasma vectors associated with natural vegetation plants pose a unique challenge, as removing them from fields involves eliminating their host plants. While this approach is more or less feasible for all three *H. obsoletus* associations, addressing *R. quinquecostatus* remains difficult. While vector species with life cycles associated with crops are likely the primary contributors to CaPsol diseases, it is unclear whether they alone are responsible for epidemic outbreaks and under what environmental conditions. This uncertainty arises from their consistent presence in crop rotation systems, while CaPsol diseases follow a recurring outbreak pattern. Crop rotation strategies designed to disrupt the link between the crops serving as hosts have been proposed as disease control measures [45,46]. However, the question arises whether ‘alternative’ vectors infiltrating the fields may be responsible for disease outbreaks that escalate after years of abating. Therefore, when investigating CaPsol occurrences, especially in epidemics, it is crucial to adopt a comprehensive approach that considers all potential vectors and their combined influence, other biological factors, as well as abiotic elements.

## 4. Materials and Methods

### 4.1. The Approach

The presented study aimed to integrate field and laboratory work to ensure comprehensive results that reflect in-field events. The experimental design was composed of two sections: (1) sampling naturally CaPsol-infected insect vectors from the planthopper family Cixiidae and (2) experimental CaPsol transmission trials to economically important plants that are threatened by this plant pathogen in Serbia and across Europe. Identification of the cixiid species involved the use of standard morphological methods with identification keys, complemented by the employment of molecular tools for precise species identification. Experimental transmission trials were conducted with selected CaPsol-infected cixiid populations to assess the vectoring potential of the chosen species in a selected crop plant, incorporating the identification and multilocus characterization of transmitted CaPsol strains in the experimental plants.

### 4.2. Field Collection of Cixiid Vectors

A survey for cixiid vectors was conducted in June and July 2022. In Rimski Šancevi (northern Serbia), the HobsUd population was found near a sugar beet field, and the HobsCa population was present on *C. arvensis* plants within the sugar beet plot and along the boundary strips. In southeastern Serbia, the HobsCf population was sampled on *C. foetida* plants along the margin of a maize field in Subotinac. Populations of *R. quinquecostatus* (Dufour) were collected from natural and semi-natural habitats to minimize or eliminate enrolment of crop plants in insect origin. Three *R. quinquecostatus* populations from northern Serbia were involved in the research: one from the *C. monogyna* shrubs in a natural habitat in Žabalj (RqCm) and two from *P. spinosa* shrubs in semi-natural habitats in Botoš (RqPs1) and Plavi Horizonti (RqPs2). In all locations, insects were collected from plants using entomological nets and mouth aspirators. Insects were initially sampled and stored in 96% ethanol at a quantity of 30 individuals for species identification and CaPsol analysis, while for the CaPsol transmission trials, insects were directly transferred to experimental plants, as further described.

### 4.3. Cixiid Vector Identification and CaPsol Detection

Identification of *H. obsoletus* (HobsUd, HobsCa, and HobsCf) was performed by examining the morphology using a Leica S9E stereomicroscope (Leica Microsystems, Wetzlar, Germany) following the identification key [47]. For *Reptalus* sp. populations found on *C. monogyna* and *P. spinosa*, it was determined if they were single species or mixed populations of *R. quinquecostatus* and *R. panzeri*, known to coexist in sympatry [11,19]. Male specimens were identified based on genital armature following the identification key [22], while female specimens were identified through molecular techniques. DNA was extracted from individual female specimens using a modified CTAB method [48]. For species-specific identification in the genus *Reptalus*, the ITS2 gene region was employed [49]. The final volume of the PCR mix (25 μL) included 1 μL of insect DNA, 1 × DreamTaq PCR Master Mix (Thermo Scientific, Vilnius, Lithuania), and 0.4 μM of ITS2fw/ITS2rv primers [50]. The PCR procedure was performed in a Biometra TOne thermal cycler (Analytik Jena AG, Jena, Germany). The obtained PCR amplicons were separated in 1% agarose gels, stained with ethidium bromide, and visualized under UV light. DNA was extracted from all insect individuals, including *H. obsoletus* males and females, as well as male *Reptalus* specimens. A total of 180 collected insect individuals, 30 per selected population (HobsUd, HobsCa, HobsCf, RqCm, RqPs1, and RqPs2), were analyzed for the presence of CaPsol. Detection was based on a CaPsol-specific *stamp* gene amplified with the Stamp-F/R0 and Stamp-F1/R1 primer pairs, following previously described conditions [51]. The HoCa430/21 CaPsol strain was used as a positive control [19].

### 4.4. Experimental Design of the CaPsol Transmission Multi-Test

Three crop plant species, sugar beet (cultivar Original), maize (cultivar NS444), and tobacco (cultivar Samsun), were enrolled as experimental plants in CaPsol transmission trials, while periwinkle plants were used as positive controls for all cixiid populations. All experimental plants were grown from seeds in mid-May, placed in pathogen-free soil, and kept in a climate chamber at 24 ± 1 °C (16/8 h light/dark period). Once all insect populations were confirmed to be CaPsol-infected, they were introduced into the trials. Sugar beet was exposed to HobsUd, the presumed vector of tuf-a/b2 CaPsol strains and *R. quinquecostatus* populations so far known to be associated with tuf-d and, for this research, collected from *C. monogyna* (RqCm) and *P. spinosa* (RqPs1 and RqPs2). Maize was exposed to HobsCa and HobsCf, both vectors of the tuf-b CaPsol type and an *R. quinquecostatus* population from *P. spinosa* (RqPs2). Tobacco was also exposed to a population of HobsCa (tuf-b type CaPsol vector) and all three studied *R. quinquecostatus* populations (RqCm, RqPs1, and RqPs2). Periwinkle plants were used as positive controls in CaPsol inoculation tests and were exposed to all six vector populations. The number of experimental plants exposed to insects depended on the abundance of the vector’s population.

Individual test plants were enclosed within plastic cylinders that allowed ventilation prior to starting transmission tests. Each experimental plant hosted 30 individuals of a specific vector population for 48 h. After the inoculation period, insects were collected and preserved in 96% ethanol. Experimental crop plants were further kept outdoors in a field net cage, while periwinkle plants were maintained in the climate chamber. Daily monitoring of symptom development was conducted during the following three months, after which the plant samples were collected. For plants that exhibited early signs of decline, samples were taken accordingly. In addition, five control plants per each plant species which were not exposed to insects were included in the experiment as negative controls.

### 4.5. Detection and Molecular Typing of CaPsol Strains in Experimental Plants

Leaf midrib tissue samples from all experimental plants underwent CTAB DNA extraction [52]. CaPsol presence in plants, as previously described for insects, was determined by amplifying the *stamp* gene using CaPsol strain 429/19 as a positive control [7]. All detected CaPsol strains underwent typing of the *tuf* and *vmp1* genes. For each PCR reaction, the final volume included 1 μL of diluted (1:50) plant DNA extract, 1× DreamTaq PCR Master Mix (Thermo Scientific, Lithuania), and 0.4 μM of each primer. The amplification of each of the three genes occurred in a corresponding nested reaction, using 1 μL of direct PCR product diluted 5 × in sterile water. Amplification of the *tuf* gene was carried out using fTuf1/rTuf1 and fTufAY/rTufAY primer pairs [12,53]. After gene amplification, an RFLP analysis for distinguishing tuf-a and tuf-b type CaPsol strains was performed utilizing the *Hpa*II enzyme (Thermo Scientific, Lithuania) according to the manufacturer’s instructions. Restriction products were separated in 8% polyacrylamide gel, stained, and visualized under UV light. Additional RFLP analyses with the *Tai*I restriction enzyme (Thermo Scientific, Lithuania) involved samples with a tuf-b profile from the previous analysis in order to distinguish between tuf-b1 and tuf-d types [7]. Moreover, samples classified as tuf-b types according to the RFLP analysis, but previously identified as tuf-a/b2 strains in *stamp* gene sequences, underwent *tuf* gene sequencing to differentiate between tuf-b1 and -b2 genotypes [13]. The *vmp1* gene was amplified in nested PCR assays using the primer pairs StolH10F1/StolH10R1 and TYPH10F/TYPH10R [54]. Nested *vmp1* amplicons underwent RFLP analysis using the *Rsa*I restriction enzyme (Thermo Scientific, Lithuania). The restriction products were separated and visualized, as previously explained. Samples with the V2 profile underwent an additional RFLP analysis using *Taq*I and *Alu*I restriction enzymes separately to determine if they contained the V2-TA type [35].

### 4.6. Genetic Relatedness of CaPsol Strains Based on the Stamp Gene

The nested *stamp* gene amplicons were sequenced commercially (Macrogen Inc. Seoul, Republic of Korea) in both directions with primers used for amplification. The obtained sequences were edited and SNPs were verified using FinchTV v. 1.4.0 (Geospiza Inc., Seattle, WA, USA) and aligned with CaPsol reference strains (reviewed in [19,20]) using ClustalX integrated into MEGA 5 software [55]. The genetic relatedness between newly described and previously known CaPsol *stamp* genotypes was assessed using a phylogenetic Median joining network constructed in NETWORK version 10.2 (www.fluxus-engineering.com) [56]. The default settings with an ε parameter value of 0 were applied, along with maximum parsimony post-processing, to create a network containing the shortest trees. This analysis included CaPsol *stamp* genotypes previously associated with the tuf-b epidemiology in Serbia.

## Figures and Tables

**Table 1:** Summary table of the CaPsol transmission multi-test. Newly detected *stamp* genotypes are labelled in bold. n.a.: not amplified.

Experimental Plant	Cixiid Vector Population	No. of CaPsol Infected/Total No. of Plants	CaPsol Genotype *tuf*/*stamp*/*vmp1* (No. of Isolates)		*Stamp* Genotype ‘St’ Code ^1^	*Stamp* Genotype Acc. No. ^2^
Sugar beet	HobsUd	4/15	tuf-a/SB5/V3	(1)	St8	
			tuf-b2/RTD6/V23	(2)	St76	
			n.a./RTD6/n.a.	(1)	St76	
	RqCm	1/5	n.a./**St96**/n.a.	(1)		OR667031
	RqPs1	7/15	tuf-b/STOL/V2-TA	(1)	St4	
			tuf-d/STOL/V2-TA	(6)	St4	
	RqPs2	3/10	tuf-d/STOL/V2-TA	(3)	St4	
Maize	HobsCa	7/15	tuf-b/STOL/V2-TA	(2)	St4	
			n.a./Rqg31/n.a.	(1)	St2	
			n.a./Rqg50/V14	(1)	St1	
			n.a./**St97**/n.a.	(1)		OR667032
			n.a./**St98**/n.a.	(1)		OR667033
			n.a./**St99**/n.a.	(1)		OR667034
	HobsCf	3/5	n.a./STOL/n.a.	(1)	St4	
			tuf-b/Rqg31/n.a.	(1)	St2	
			n.a./**St100**/V14	(1)		OR667035
	RqPs2	4/10	tuf-b/STOL/V2-TA	(1)	St4	
			n.a./STOL/n.a.	(2)	St4	
			n.a./**St101**/n.a.	(1)		OR667036
Tobacco	HobsCa	8/10	tuf-b/Rqg31/V2-TA	(3)	St2	
			tuf-b/Rqg31/V4	(1)	St2	
			tuf-b/Rqg31/V14	(2)	St2	
			tuf-b/Rqg50/V14	(1)	St1	
			n.a./**St102**/n.a.	(1)		OR667037
	RqCm	2/5	tuf-b/Rqg31/n.a.	(1)	St2	
			tuf-d/STOL/V2-TA	(1)	St4	
	RqPs1	2/5	tuf-b/STOL/V2-TA	(1)	St4	
			tuf-d/STOL/V2-TA	(1)	St4	
	RqPs2	3/5	tuf-b/Rqg31/V2-TA	(1)	St2	
			tuf-d/STOL/V2-TA	(2)	St4	
Periwinkle	HobsUd	5/5	tuf-b2/19-25/V18	(2)	St11	
			tuf-b2/RTD6/V23	(3)	St76	
	HobsCa	5/5	tuf-b/Rqg31/V14	(2)	St2	
			tuf-b/Rpm35/V14	(2)	St3	
			tuf-d/STOL/V2-TA	(1)	St4	
	HobsCf	5/5	tuf-b/STOL/V2-TA	(3)	St4	
			tuf-b/Rqg50/V14	(2)	St1	
	RqCm	5/5	tuf-b/STOL/V2-TA	(2)	St4	
			tuf-d/STOL/n.a.	(3)	St4	
	RqPs1	5/5	tuf-b/Rqg50/V4	(1)	St1	
			tuf-d/STOL/V2-TA	(4)	St4	
	RqPs2	5/5	tuf-b/Rqg50/V4	(1)	St1	
			tuf-d/STOL/V2-TA	(4)	St4	

^1^ The ‘St’ stamp sequence variants corresponding to the original *stamp* genotype designation [19,33,34]; ^2^ accession numbers are provided for the newly described *stamp* genotypes.

## Data Availability

DNA sequences of the *stamp* gene are available in the GenBank database; accession numbers are given in Table 1 in the Results section. All other relevant data are within the paper.
